# The Green Berry Consortia of the Sippewissett Salt Marsh: Millimeter-Sized Aggregates of Diazotrophic Unicellular Cyanobacteria

**DOI:** 10.3389/fmicb.2017.01623

**Published:** 2017-09-04

**Authors:** Elizabeth G. Wilbanks, Verena Salman-Carvalho, Ulrike Jaekel, Parris T. Humphrey, Jonathan A. Eisen, Daniel H. Buckley, Stephen H. Zinder

**Affiliations:** ^1^Department of Ecology, Evolution, and Marine Biology, University of California, Santa Barbara, Santa Barbara CA, United States; ^2^HGF MPG Joint Research Group for Deep Sea Ecology and Technology, Max Planck Institute for Marine Microbiology Bremen, Germany; ^3^Department for Microbiology, Max Planck Institute for Marine Microbiology Bremen, Germany; ^4^Department of Organismic and Evolutionary Biology, Harvard University, Cambridge MA, United States; ^5^Genome Center, Department of Evolution and Ecology, Department of Medical Microbiology and Immunology, University of California, Davis, Davis CA, United States; ^6^School of Integrative Plant Science, Cornell University, Ithaca NY, United States; ^7^Department of Microbiology, Cornell University, Ithaca NY, United States

**Keywords:** nitrogen fixation, cyanobacteria, UCYN-A, UCYN-B, biofilms, marine aggregate, unicellular cyanobacteria, salt marsh

## Abstract

Microbial interactions driving key biogeochemical fluxes often occur within multispecies consortia that form spatially heterogeneous microenvironments. Here, we describe the “green berry” consortia of the Sippewissett salt marsh (Falmouth, MA, United States): millimeter-sized aggregates dominated by an uncultured, diazotrophic unicellular cyanobacterium of the order *Chroococcales* (termed GB-CYN1). We show that GB-CYN1 is closely related to *Crocosphaera watsonii* (UCYN-B) and “*Candidatus* Atelocyanobacterium thalassa” (UCYN-A), two groups of unicellular diazotrophic cyanobacteria that play an important role in marine primary production. Other green berry consortium members include pennate diatoms and putative heterotrophic bacteria from the *Alphaproteobacteria* and *Bacteroidetes*. Tight coupling was observed between photosynthetic oxygen production and heterotrophic respiration. When illuminated, the green berries became supersaturated with oxygen. From the metagenome, we observed that GB-CYN1 encodes photosystem II genes and thus has the metabolic potential for oxygen production unlike UCYN-A. In darkness, respiratory activity rapidly depleted oxygen creating anoxia within the aggregates. Metagenomic data revealed a suite of nitrogen fixation genes encoded by GB-CYN1, and nitrogenase activity was confirmed at the whole-aggregate level by acetylene reduction assays. Metagenome reads homologous to marker genes for denitrification were observed and suggest that heterotrophic denitrifiers might co-occur in the green berries, although the physiology and activity of facultative anaerobes in these aggregates remains uncharacterized. Nitrogen fixation in the surface ocean was long thought to be driven by filamentous cyanobacterial aggregates, though recent work has demonstrated the importance of unicellular diazotrophic cyanobacteria (UCYN) from the order *Chroococcales.* The green berries serve as a useful contrast to studies of open ocean UCYN and may provide a tractable model system to investigate microbial dynamics within phytoplankton aggregates, a phenomenon of global importance to the flux of particulate organic carbon and nitrogen in surface waters.

## Introduction

Fixed nitrogen is often a limiting nutrient for primary productivity in the surface ocean, and consequently influences the dynamics of oceanic carbon sequestration (Karl et al., 2002). Nitrogen (N_2_) fixation by marine cyanobacteria is an important source of oceanic fixed nitrogen, adding an estimated 100–200 Tg-N annually to open ocean ecosystems (Karl et al., 2002; Galloway et al., 2004). This nitrogen fixation is often associated with cyanobacterial trichomes or aggregates colonized by heterotrophic bacteria, picoeukaryotes and metazoans (Paerl et al., 1989; Hewson et al., 2009; Ploug et al., 2010). Respiratory activity within these so-called ‘pseudo-benthic’ environments can create ephemeral suboxic to anoxic zones, establishing a niche for facultative anaerobes within otherwise oxygenated surface waters (Paerl and Prufert, 1987; Ploug et al., 2011; Klawonn et al., 2015). Emerging evidence suggests that denitrification occurs within these anoxic habitats, coupling processes of nitrogen-fixation and loss at the microscale (Ploug et al., 2011; Wyman et al., 2013; Klawonn et al., 2015).

While initial studies of marine biological nitrogen fixation focused on colonial filamentous *Trichodesmium* species (Capone et al., 1997) and symbiotic, heterocystous *Richelia* species (Foster and Zehr, 2006), more recent work has demonstrated the importance of unicellular diazotrophic cyanobacteria (UCYN) from the order *Chroococcales* (Montoya et al., 2004; Zehr et al., 2007). Diazotrophic UCYN have been studied extensively in the global oceans by surveys of the nitrogenase gene *nifH* diversity, which revealed three phylogenetically distinct clades (A-C) (Zehr et al., 2001; Langlois et al., 2005; Foster et al., 2007). UCYN-A are small (*circa* 1 μm), metabolically streamlined, uncultured cyanobacteria that lack the oxygen-producing photosystem II and live as endosymbionts within haptophytes, a lineage of eukaryotic algae (Zehr et al., 2008; Tripp et al., 2010; Thompson et al., 2012; Hagino et al., 2013). UCYN clades B and C are larger (>2 μm), free-living cyanobacteria and include cultured representatives, such as *Crocosphaera watsonii* and *Cyanothece* sp. ATCC51142.

Studies of aggregate-associated nitrogen fixation have focused predominantly on *Trichodesmium* sp. colonies and rafts (Paerl et al., 1989), or filamentous heterocystous cyanobacterial colonies (Ploug, 2008; Ploug et al., 2010; Klawonn et al., 2015). However, some *Crocosphaera watsonii* strains have been observed to produce copious quantities of exopolysaccharides and have been linked to the formation of transparent exopolymer particles (TEP) (Webb et al., 2009; Sohm et al., 2011). These gel-like particles provide microhabitats for other microorganisms, and thus have the potential to play an important role in marine biogeochemical cycling (Passow, 2002).

Here, we report a new species of uncultured, unicellular cyanobacteria from the order *Chroococcales* which forms millimeter-sized aggregates together with diatoms and other putatively heterotrophic bacteria. These macroscopic aggregates, which we call “green berries,” are found in the muddy, intertidal pools of Little and Great Sippewissett salt marshes (Falmouth, MA, United States). They are found interspersed with previously described, sulfur-cycling “pink berry” consortia (Seitz et al., 1993; Wilbanks et al., 2014). Using a combination of metagenomic sequencing and ecophysiological measurements, we demonstrate that the green berries are characterized by diazotrophy and rapid rates of photosynthesis and respiration that produce steep oxygen gradients. Heterotrophic bacteria within the green berries are closely related to other marine epiphytic marine strains and encode key genes in the denitrification pathway.

## Results and Discussion

### Morphological Description of the Aggregates

**FIGURE 1:**
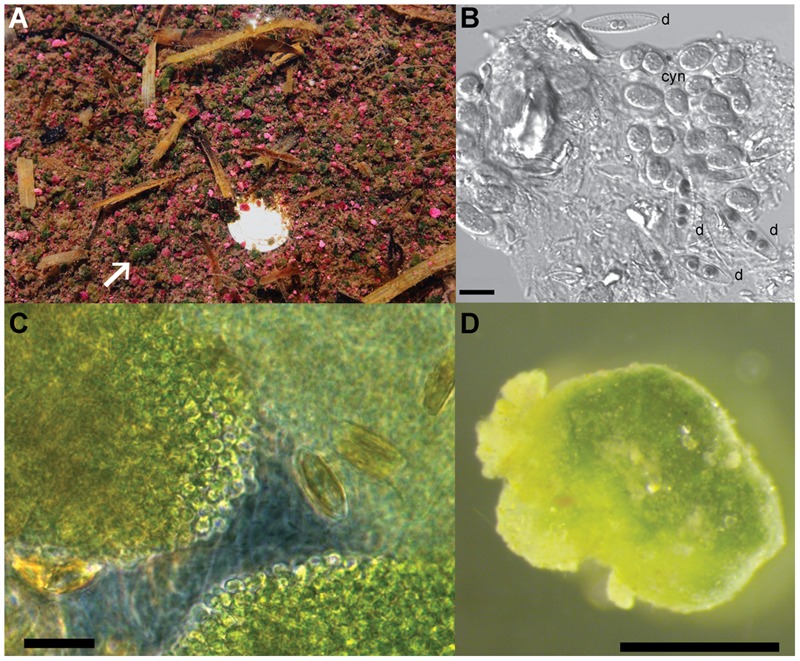
Morphology of the green berries. **(A)** Pink and green berries at the sediment water interface of intertidal pools in the Little Sippewissett salt marsh. White arrow indicates a large green berry and a dime (bright spot) is provided for scale. **(B)** Differential interference contrast (DIC) micrograph of cells from a homogenized green berry showing two different pennate diatom morphotypes (d) and clumps of coccoid unicellular cyanobacteria 5–7 μm in diameter (cyn). Scale bar = 10 μm. **(C)** Phase contrast image of an intact green berry aggregate compressed under a coverslip. Note the dense clumps of coccoid cyanobacterial cells and pennate diatoms interspersed in a clear exopolymer matrix. Scale bar = 10 μm. **(D)** Dissecting microscope image of a single green berry aggregates. Scale bar = 1 mm.

The green berries are found in the same organic-rich, intertidal pools of Little Sippewissett salt marsh on Cape Cod (MA, United States) where both multicellular magnetotactic bacteria and pink berries have been previously studied (Seitz et al., 1993; Shapiro et al., 2011; Wilbanks et al., 2014). Though less abundant than the pink berries found in these pools (**Figure 1A**), the green berries form similar irregular ellipsoid aggregates measuring 1–8 mm in diameter, with an average equivalent spherical diameter of 1.7 mm ± 0.1 mm (standard deviation, **Figures 1A,D**). Green berries were dense and compact aggregates that were typically observed at the sediment-water interface, but were occasionally found to float at the water surface when suspended by bubbles. Microscopic observation of the green berries revealed abundant coccoid unicellular cyanobacteria 5–7 μm in diameter (which we call GB-CYN1, “green berry cyanobacteria 1”), interspersed with pennate diatoms (**Figures 1B,C**). Filamentous cyanobacteria were observed occasionally, but were rare compared to the unicellular GB-CYN1 morphotype. A clear, extracellular matrix (putatively exopolysaccharides) coated these aggregates of phototrophic cells, and was colonized by a variety of smaller bacteria (**Figures 1B,C**). GB-CYN1 exhibited absorption maxima at 620, 660, and 680 nm corresponding to the presence of phycocyanin, allophycocyanin and chlorophyll *a*, respectively.

### Microbial Diversity of the Green Berry Aggregates

Sequencing of 18S rRNA genes from the green berries indicated that the eukaryotic community was predominantly made up of two different pennate diatom species related to *Navicula cari* strain AT-82.04c (96% sequence identity) and *Amphora pediculus* strain AT-117.11 (95% sequence identity; Supplementary Figure S1). These same diatom species were also the dominant eukaryotic 18S rRNA gene sequences recovered from pink berry aggregates, though diatoms were more abundant in green berries than in pink berries, as observed by microscopy and the relative abundance of 16S rRNA chloroplast sequences (Wilbanks et al., 2014).

**FIGURE 2:**
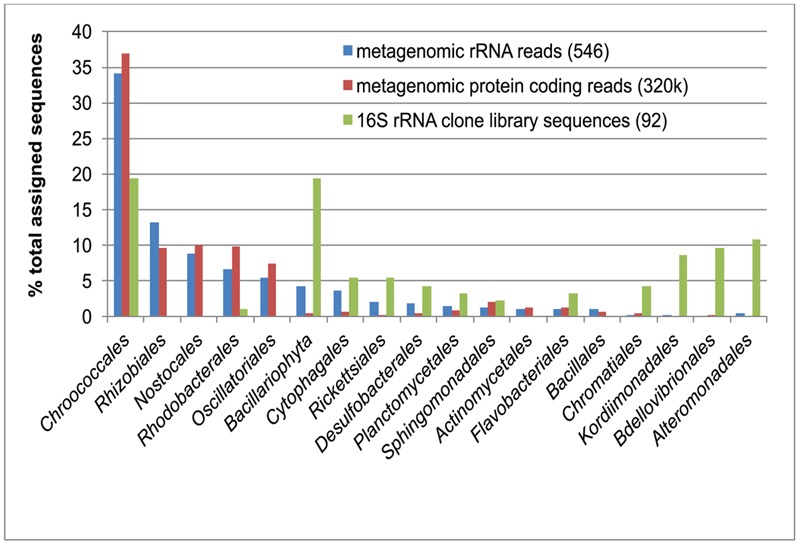
Comparison of green berry bacterial diversity estimates from16S rDNA PCR amplified clones library (green bars; 92 clone sequences) with unassembled Roche 454 metagenomic sequence reads. Taxonomic assignment of metagenomic reads matching ribosomal RNA reads (16S/18S/23S) was conducted using the M5RNA database in MG-RAST (blue bars; 546 assigned reads). A similar taxonomic assignment was conducted with metagenomic reads matching protein coding sequences in the M5NR database (red bars; 319,692 assigned reads). Note that the 16S rRNA clone library abundance data for the *Bacillariophyta* (green bar, asterisk) was obtained from diatom chloroplasts sequences, which are likely present in multiple copies in the cell and thus not directly comparable to metagenomic 18S rRNA sequences for this group (blue bar).

Bacterial 16S rRNA gene sequences amplified from the green berries were dominated by sequences related to either diatom chloroplasts (phylum *Bacillariophyta*, 18/92 clones, representing 4 OTUs at 97% similarity threshold) or *Chroococcales* unicellular cyanobacteria (18/92 clones, representing 2 OTUs; **Figure 2**). Unassembled metagenomic sequence reads assigned to rRNA sequences and protein-coding regions support the observed abundance of *Chroococcales* (accounting for up to 37% relative sequence abundance), but did not recover comparable proportions of diatom chloroplasts (<5% relative sequence abundance; **Figure 2**).

The overall bacterial community structure of the green berries was significantly different from coexisting pink berry consortia (unweighted UniFrac analysis, Bonferroni corrected *p*-value < 0.01). Some abundant taxa from the pink berries co-occurred in the green berries as rare OTUs, such as the purple sulfur bacterial species *Thiohalocapsa* sp. PB-PSB1 (1/92 clones), and a *Winogradskyella* species (*Flavobacteriales*, 2/92 clones) (Wilbanks et al., 2014). The persistence of these distinct, co-occurring pink and green berry consortia suggests that the process of macroscopic aggregation enables niche partitioning between oxygenic (green berries) and anoxygenic (pink berries) phototrophs in these marsh pools.

Most of the non-cyanobacterial sequences in the green berry consortia are related to aerobic and facultatively anaerobic marine heterotrophs from the *Bacteroidetes, Alphaproteobacteria*, and *Gammaproteobacteria* (**Figure 2**). Many of these sequences (e.g., taxa from the *Rhodobacterales, Kordiimonadales, Sphingomonadales*, and *Flavobacterales*) were most closely related to environmental 16S rRNA sequences associated with aggregates of oxygenic phototrophs. Examples of such habitats included phytodetrital aggregates (marine snow) collected from euphotic and hadal environments (DeLong et al., 1993; Eloe et al., 2011), and epiphytes of marine macroalgae (Burke et al., 2011; Fernandes et al., 2012). The occurrence of related phylotypes in such environments suggests that taxa may be well adapted to an attached lifestyle, degradation of photosynthate, and the fluctuating oxygen conditions in an aggregate environment.

Metagenomic data indicate that the orders *Rhizobiales* and *Rhodobacterales* of the *Alphaproteobacteria* are abundant in the green berry consortia. While these groups were rarely detected in the PCR-based 16S rRNA survey, we have previously observed this same PCR bias from the 8F primer during studies of the pink berry consortia (Wilbanks et al., 2014). We find the abundance of these clades in the green berries particularly interesting as they include lineages of marine denitrifying bacteria. For example, pelagic *Rhizobiales* (e.g., *Labrenzia* and *Roseibium* species) have been linked to denitrification when found in association with macroscopic *Trichodesmium* sp. aggregates in oxic waters bordering oxygen minimum zones (Wyman et al., 2013).

### Phylogenomic Analysis of the Green Berry Cyanobacteria, GB-CYN1

**FIGURE 3:**
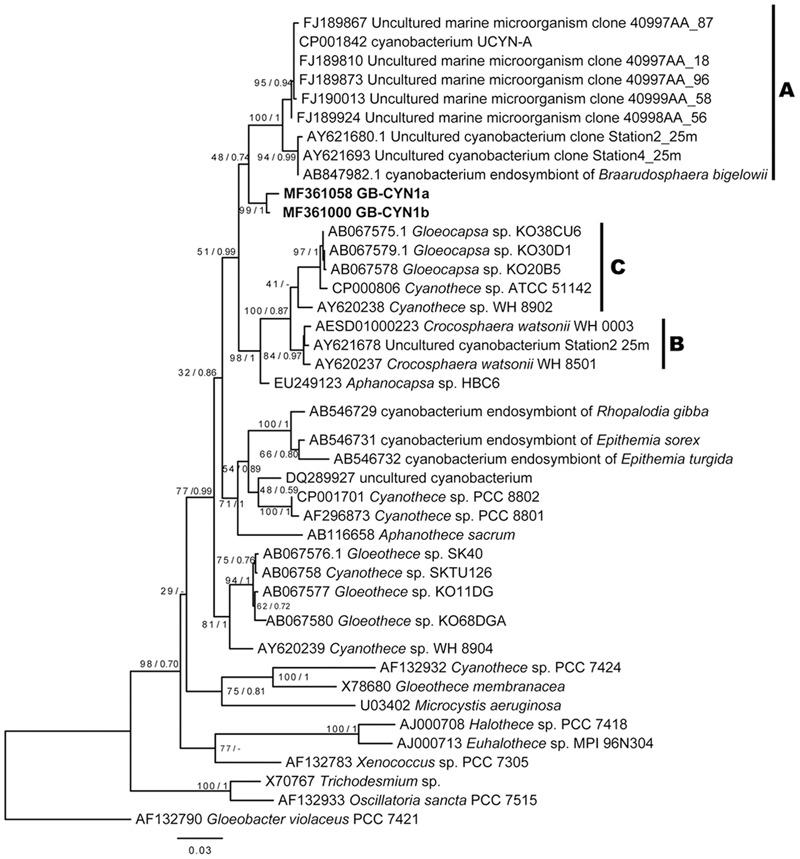
Maximum likelihood and Bayesian 16S rRNA gene phylogenies of GB-CYN1 species and related cyanobacteria. Reference sequences were full length and GB-CYN1 OTUs (bold) were partial (∼700–800 bp). Constructed from (A) 16S rRNA sequences from (partial sequences, ∼700–800 bp, bold) and Numbers at the nodes indicate bootstrap values from maximum likelihood analysis (1000 replicates) and the posterior probability of the Bayesian tree inference. The Bayesian consensus tree was concordant with maximum likelihood tree topology except where indicated by a dash. Scale bar represents the mean number of nucleotide substitutions per site. The major clades of unicellular cyanobacteria (UCYN-A-C) are labeled according to (Zehr et al., 2001; Langlois et al., 2005; Foster et al., 2007).

The cyanobacterial 16S rRNA gene sequences from the green berries grouped into two closely related OTUs (97% similarity threshold), GB-CYN1a and GB-CYN1b, that can be confidently placed in the order *Chroococcales* (**Figure 3**). The GB-CYN1 monophyletic cluster formed a clade basal to the UCYN-A clade (96% sequence identity to CP001842, *“Candidatus* Atelocyanobacterium thalassa” isolate ALOHA). Using 29 concatenated single-copy phylogenetic marker genes (Wu et al., 2013) assembled from the metagenome (**Supplementary Table S1**), we reconstructed a phylogenetic tree that placed the GB-CYN1 within a clade including *Crocosphaera watsonii* and *Cyanothece* sp. ATCC 51142 as a sister taxa to *“Candidatus* Atelocyanobacterium thalassa” isolate ALOHA (Supplementary Figure S2).

**FIGURE 4:**
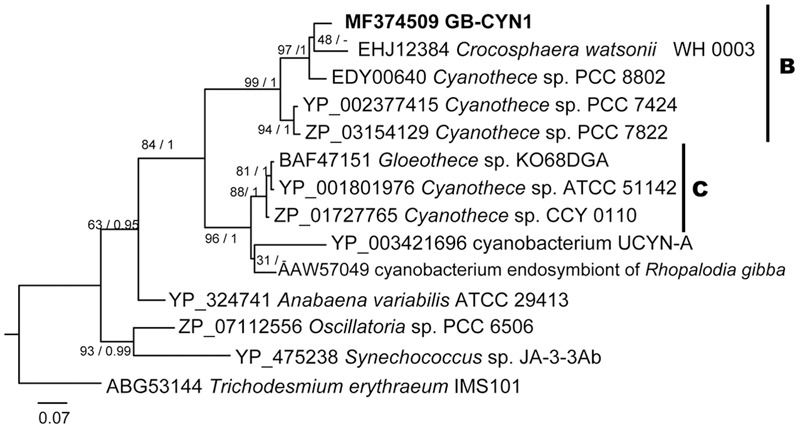
Maximum likelihood and Bayesian NifH phylogenies of GB-CYN1 species and related cyanobacteria. Near-full length NifH amino acid sequence from GB-CYN1 (267aa, from assembled metagenomic contig) was aligned with full length reference sequences. Numbers at the nodes indicate bootstrap values from maximum likelihood analysis (1000 replicates) and the posterior probability of the Bayesian tree inference. The Bayesian consensus tree was concordant with maximum likelihood tree topology except where indicated by a dash. Scale bar represents the mean number of amino acid substitutions per site. The major clades of unicellular cyanobacteria (UCYN-A-C) are labeled according to Zehr et al. (2001), Langlois et al. (2005), and Foster et al. (2007).

A phylogenetic tree inferred from *nifH* gene sequences reveals that the near full-length *nifH* gene recovered from the GB-CYN1 metagenomic data affiliated with the UCYN-B clade, and was most closely related to *Cyanothece* sp. 8801/8802 and *Crocosphaera watsonii* (**Figure 4**). We conclude that the observed discordance between 16S rRNA, concatenated, and *nifH* gene phylogenies involving species such as *Cyanothece* sp. 8801, *Gloeothece* sp. KO68DGA, and the cyanobacterial endosymbiont of *Rhopalodia gibba* is most likely due to lateral gene transfer of the *nifH* gene. Lateral transfer of *nifH* has been observed in many other species, including mat-forming filamentous cyanobacteria (Cantera et al., 2004; Bolhuis et al., 2010; Parker, 2012; Gaby and Buckley, 2014; Meyer and Huber, 2014).

### Diazotrophy in the Green Berries

A full suite of nitrogenase genes were found in the green berry metagenome and were consistently assigned to GB-CYN1, indicating the metabolic potential for nitrogen fixation characteristic of other members of the UCYN A-C clades (**Supplementary Table S1**). Diazotrophy in the green berries was detected in whole aggregates by acetylene reduction. In two separate experiments (containing 5 berries each), we measured rates of 11 and 20 nanomoles acetylene reduced per hour per milligram of aggregate dry weight (nmol mg^-1^ hr^-1^). These rates are comparable, though faster than the rates of 3–6 nmol mg^-1^ hr^-1^ reported in macroscopic aggregates of filamentous cyanobacteria from Bogue Sound, North Carolina (Paerl and Prufert, 1987). Rates measured from actively growing *Cyanothece* cultures (Reddy et al., 1993) were two orders of magnitude larger (∼1000 nmol mg^-1^ hr^-1^) than the rates in the green berries.

The total aggregate elemental composition was analyzed using elemental analyzer isotope ratio mass spectrometry (EA-IRMS). The mean observed C:N ratio in the green berries, 7.1 ± 0.6 (*n* = 6), falls within range of the Redfield molar ratio (Redfield et al., 1963). This observed C:N ratio is higher than that the ratio of 5.4 ± 0.4 (*n* = 50) observed in similarly large, anoxic, diazotrophic *Nodularia spumigena* aggregates from the Baltic Sea (Klawonn et al., 2015). Cultures of *Crocosphaera watsonii* exhibit wide diel fluctuations in C:N ratios (∼5 at dawn to ∼9 at dusk) as a result of temporal partitioning of carbon (day) and nitrogen (night) fixation activities (Dron et al., 2012, 2013). Our samples, collected in the late afternoon on a 14 h light/10 h dark photoperiod, are comparable to reports of C:N = 7 from *C. wastsonii* at similar late afternoon times in a 16 h light/8 h dark photoperiod (Dron et al., 2013). Future studies investigating the temporal partitioning for such activities in the green berry aggregate would be informative to elucidate the dynamics of carbon and nitrogen flow in the consortia.

### Respiration and Photosynthesis

**FIGURE 5:**
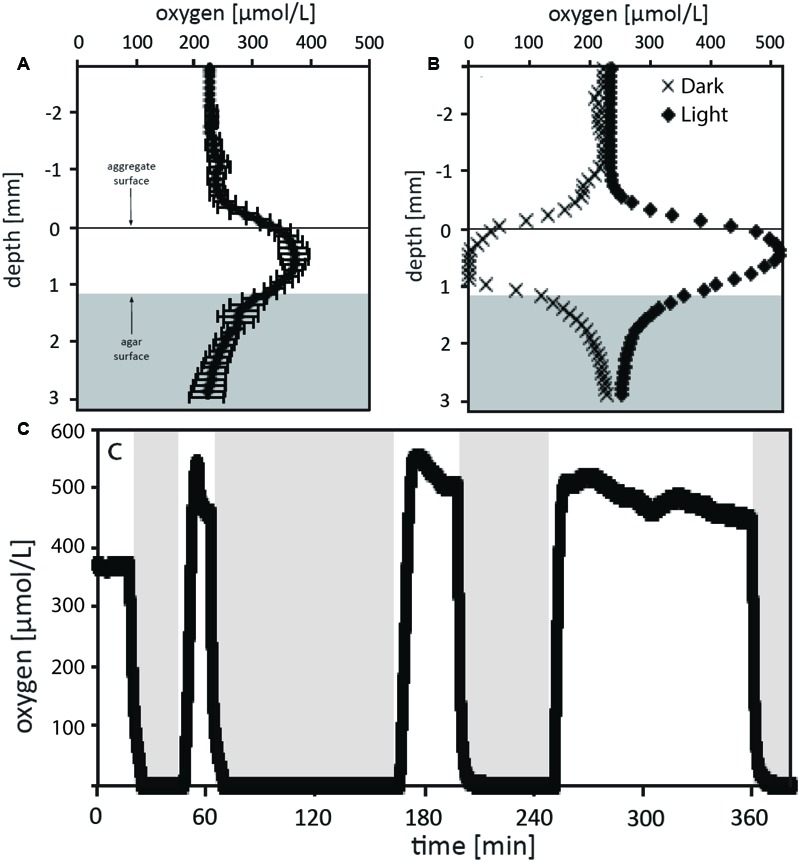
Oxygen measurements of the green berry aggregates in light and dark conditions. **(A)** Depth microprofiles with oxygen microsensors were conducted for 3 similar aggregates under steady state illumination with 170 μE m^-2^ s^-1^. The average O_2_ concentration versus depth relative to aggregate surface is plotted for these aggregates. **(B)** O_2_ profiles were measured for an aggregate under conditions of steady state illumination at 320 μE m^-2^ s^-1^ (black diamonds) and steady state darkness (black Xs). **(C)** The O_2_ microsensor was positioned at the center of an aggregate which was then exposed to series of different illumination conditions over a time course while concentration was monitored. Initial conditions recorded were at 170 μE m^-2^ s^-1^ followed by shift to darkness, and three subsequence rounds of illumination at 320 μE m^-2^ s^-1^ followed by dark shifts. Dark periods are indicated by the position of the light gray boxes.

Oxygen microsensors were used to characterize the balance between respiration and photosynthesis in the green berry aggregates. Aggregates examined were relatively symmetric ellipsoids of similar size with an equivalent spherical diameter (ESD) of 1.7 ± 0.1 mm (*n* = 5, average ± standard deviation). Photosynthesis produced supersaturated oxygen concentrations within the aggregates: 380 μM O_2_ with illumination at 170 μE m^-2^ s^-1^ (one lamp) and 520 μM O_2_ at 320 μE m^-2^ s^-1^ (two lamps; **Figure 5**). During these experiments, bubbles were not observed on aggregate surfaces and the aggregates never floated. Oxygen production rates per aggregate (net photosynthesis) were calculated from these profiles as 13 and 31 nmol O_2_ per hour at 170 μE m^-2^ s^-1^ and 320 μE m^-2^ s^-1^, respectively (**Table 1**). We did not rigorously determine the saturating light intensity; however, we observed that illumination with a third lamp failed to stimulate increased oxygen production beyond that with two lamps (320 μE m^-2^ s^-1^), suggesting that the saturating light intensity lies in the range of 170 – 320 μE m^-2^ s^-1^ (data not shown).

**Table 1:** Rates of respiration and photosynthesis for green berries.

Light level μE m^-2^ s^-1^	Dark Resp. nmol h^-1^	Vol. Dark Resp μmol cm^-3^ h^-1^	Net Phot. nmol h^-1^	Vol. Net Phot. μmol cm^-3^ h^-1^	Gross Phot. nmol h^-1^	Vol. Gross Phot. μmol cm^-3^ h^-1^	L-D shift Vol. Gross Phot μmol cm^-3^ h^-1^	Carbon fixation ng h^-1^
0	19	6.8	–	–	–	–	–	–
170	–	–	13	4.8	32	11.6	7.2	320
320	–	–	31	11	50	18	13.5	496

*Rates were calculated from oxygen depth microprofiles in the light and dark (according to Ploug et al., 1997), with the exception of final column which calculated the volumetric rate of gross photosynthesis at the aggregate core according to the light-dark shift method (Revsbech et al., 1981). Carbon fixation was estimated based on a photosynthetic quotient of 1.2 (Masotti et al., 2007).*

The green berries were anoxic in darkness with a dark respiration rate of 19 nmol per aggregate per hour (**Figure 5B**). This rate, equivalent to a volumetric rate of 6.8 μmol cm^-3^ hr^-1^, is well above the theoretical threshold for respiration rates capable of causing oxygen diffusional limitation from surrounding seawater (5.0 μmol cm^-3^ hr^-1^ for ESD = 1.7 mm; calculated after Ploug et al., 1997). In light-dark shifts, we observed a rapid response where the aggregate core transitioned from steady state supersaturation to full anoxia in 5–8 min (**Figure 5C**).

Volumetric gross photosynthetic rates were calculated by two methods: (1) from depth microprofiles via the sum of net photosynthesis and dark respiration and (2) via the light-dark shift technique (Revsbech et al., 1981) performed at a single point in the aggregate center (**Table 1**). At both light intensities examined, rates calculated via the light-dark shift method were found to be 4.5 μmol cm^-3^ hr^-1^, lower than those from depth microprofiles. While this difference could arise from biological variability between aggregates, we suspect that the light-dark shift rates measured at the aggregate core were lower than those we might have measured closer to the aggregate surface. Future depth integrated studies of photosynthetic rates will help to clarify this difference and allow better characterization of respiratory activity in the light.

Comparing the green berries’ dark respiration and gross photosynthesis to other photosynthetic mats and aggregates, we find them similar to the high rates measured for large (>1 mm), filamentous aggregates of the heterocystous cyanobacterium, *Nodularia spumigena* from the Baltic Sea (Ploug et al., 2011). Indeed, our estimates of carbon fixation (**Table 1**) are close to prediction of 349 ng C per aggregate per hour calculated using Ploug et al.’s (2011) regression of volume to gross photosynthesis from a 2009 *Nodularia* bloom. The green berry dark respiration rate (19 nmol agg^-1^ h^-1^) was similar, though slightly higher than that observed for *Nodularia* aggregates of similar diameter; however, the ratio of dark respiration to gross photosynthesis of 0.38 (for an aggregate volume of 2.8 mm^3^) was identical to that observed for 7 mm^3^ aggregates found late in the bloom (Ploug et al., 2011). In large *Nodularia* aggregates, dark anoxia is associated with active dissimilatory nitrogen cycling, including denitrification, dissimilatory reduction of nitrate to ammonia (DNRA), and significant rates of cryptic nitrification (Klawonn et al., 2015).

### Ecophysiological Implications from Metagenome Data

Our metagenomic data analysis predicts genes in GB-CYN1 involved in the Calvin cycle, TCA cycle, and photosystems I and II (**Supplementary Table S1**). Sequence identities for these genes in GB-CYN1 and published *Chroococcales* genomes were high, ranging from 75 to 99% (average of 85% for assembled contigs, **Supplementary Table S1**). The observation of photosystem II genes in GB-CYN1 indicates the metabolic potential for oxygenic photosynthesis, unlike UCYN-A which lacks photosystem II, RuBisCo and the TCA cycle. These findings confirm that GB-CYN1 resembles free-living *Chroococcales* of the clades B and C (Welsh et al., 2008; Bandyopadhyay et al., 2011; Bench et al., 2011, 2013) rather than the metabolically streamlined, endosymbiotic UCYN-A clade (Zehr et al., 2008; Tripp et al., 2010; Thompson et al., 2012; Hagino et al., 2013).

Related *Chroococcales* species are known to produce copious quantities of exopolysaccharides (EPS) (Pereira et al., 2009; Sohm et al., 2011; Mota et al., 2013), and EPS produced by *Crocosphaera watsonii* has been linked to the formation of transparent exopolymeric particles in pelagic environments (Passow, 2002; Webb et al., 2009; Sohm et al., 2011). A cassette of genes required for EPS production has been identified by comparative genomic analysis of related *Crocosphaera watsonii* strains (Webb et al., 2009; Bench et al., 2011, 2013); however, we did not recover homologs of these genes in our metagenomic sequence (either assembled contigs or sequence reads). We conclude that the absence of these sequences is most likely due to our incomplete sampling of the GB-CYN1 genome, though it could also indicate either an unknown pathway for EPS synthesis in GB-CYN1, or an alternate source of the green berry exopolymeric matrix (e.g., diatoms).

Consistent with the dynamic oxygen conditions within the green berries, we found sequences in the metagenomic data that suggest the metabolic potential for aerobic and anaerobic respiration, and anaerobic fermentative pathways assigned to several different phyla of bacteria (data not shown). Abundant fixed nitrogen from diazotrophy and transient anoxia presents an ecological opportunity for dissimilatory nitrogen metabolism, a process that could cause concomitant nitrogen fixation and loss over rapid spatiotemporal scales. Indeed, previous studies of diazotrophic *Nodularia spumigena* aggregates measured significant rates of both nitrification and denitrification (Klawonn et al., 2015).

We specifically investigated potential marker genes for dissimilatory nitrogen metabolism within the green berries. Metagenomic sequences homologous to the nitrite reductase gene *nirK* were found in six unassembled reads and on a corresponding 753 bp long contig (**Supplementary Table S2**). A single read homologous to nitrous oxide reductase *nosZ* was also found suggesting the presence of a denitrification pathway (**Supplementary Table S2**). The top database matches to these *nirK* and *nosZ* sequences belonged to marine phytoplankton epiphytes from the *Alphaproteobacteria* (*nirK*) and *Flavobacterales* (*nosZ*). In the case of the *nirK* metagenomic sequences, the best match was to *Roseibium* sp. TrichSKD4, an alphaproteobacterial species isolated from a nitrogen-fixing *Trichodesmium* aggregate in the Atlantic Ocean (Mann and Barbeau, 2014). Homologs to the *napA* periplasmic nitrate reductase and the *nirB* nitrite reductase were identified from six and five unassembled reads, respectively, and were most similar to database sequences from marine heterotrophs in the *Alphaproteobacteria (Rhodobacterales, Rhizobiales*) and *Gammaproteobacteria* (*Oceanospirillales*; **Supplementary Table S2**). We did not detect sequences supporting the presence of nitrifying bacteria or archaea, though given our limited sequence depth, this could be a function of missing data.

## Conclusion

Cyanobacterial nitrogen fixation in the global oceans is frequently aggregate-associated, as with *Trichodesmium* sp. colonies and rafts (Paerl et al., 1989), filamentous heterocystous cyanobacterial colonies (Ploug, 2008; Ploug et al., 2010), or *Crocosphaera watsonii* associated with TEP (Webb et al., 2009; Sohm et al., 2011). The green berries of the Sippewissett Salt Marsh are nitrogen-fixing macroscopic consortia of unicellular cyanobacteria (GB-CYN1), diatoms and heterotrophic bacteria. While nitrogen and carbon fixation mediated by the green berries is unlikely to play a major role in the overall marsh ecosystem due to their low abundance and patchy distribution (Carpenter et al., 1978; Howarth et al., 1988), these consortia provide an interesting comparative system to investigate the dynamics nitrogen flux within diazotrophic cyanobacterial aggregates. More broadly, studies of coastal marine estuarine sediments have indicated complex dynamics and close spatial coupling of co-occurring nitrogen fixation and denitrification processes (Fulweiler et al., 2013).

In other oceanic diazotrophic cyanobacterial aggregates, similarly rapid respiratory rates create transient anoxic zones within the aggregate core (Paerl and Bebout, 1988; Ploug et al., 2011; Klawonn et al., 2015), and create a heterogeneous microenvironment where both aerobic and anaerobic metabolisms co-exist. This parallel is not merely conceptual: the green berry heterotrophic bacteria were similar, both phylogenetically and in their metabolic marker genes, to those observed to colonize other marine phytoplankton aggregates. The recovery of denitrification marker gene sequences in metagenome suggests that there might be heterotrophic denitrifiers in the green berries with the potential to exploit this suboxic niche. However, further studies including rate measurements and better genome reconstructions are needed to clarify the importance of denitrification in the berries.

The presence of such a pathway for nitrogen loss in the green berries is speculative, given the fragmented metagenome and absence of activity measurements, but remains an interesting direction for future work. The existence of closely coupled nitrogen cycling within diazotrophic cyanobacterial aggregates has been explored previously, though initial studies demonstrating the association of heterocystous cyanobacterial aggregates with key bacterial species and marker genes for nitrification and denitrification measured only low to negligible rates (Hietanen et al., 2002; Tuomainen et al., 2003, 2006). However, more recent investigations of *in vitro* rates and *in situ* marker gene transcription indicates that denitrification within anoxic cyanobacterial aggregates could contribute significantly to nitrogen loss in hypoxic waters, where bulk oxygen concentrations would normally inhibit such activity (Wyman et al., 2013; Klawonn et al., 2015). In the light of modeling studies showing tight spatiotemporal coupling of nitrogen fixation and denitrification processes in the global ocean (Deutsch et al., 2007), we postulate that this coupling may be occurring at the microscale within ephemeral, aggregate-associated niches. The potential for such interactions emphasizes the need to examine biogeochemical cycles from the microbial perspective (nanometers to millimeters) in the spatially heterogeneous marine environment.

## Experimental Procedures

### Sampling and Microscopy

Green berries were sampled in June and July of 2010 (for all studies except O_2_ microsensor work) or July and August 2014 (for oxygen microsensors) from a single intertidal pool formed in the Little Sippewissett Salt Marsh, Falmouth, MA United States (41°34′33.01″N, 70°38′21.24″W). These aggregates, however, have also been observed at other locations throughout Little and Great Sippewissett marshes. Berries were collected from the sediment-water interface by sieving (1 mm mesh size) and were washed three times in 0.2 μm filter sterilized marsh water. Imaging of the berries was conducted using a Zeiss Axio IMAGER MZ epifluorescence microscope equipped with a color camera (AxioCam HRc, Zeiss) and a Zeiss LSM 710 spectral confocal scanning laser microscope (Carl Zeiss, Oberkochen, Germany).

### DNA Sequencing and Assembly

DNA extraction, 16S/18S rRNA PCR, and clone libraries were performed as described previously (Wilbanks et al., 2014). Roche GC Titanium 454 metagenomic sequencing was performed as described previously (Wilbanks et al., 2014), generating 100 megabases (Mbp) of sequence data after quality filtering (275,263 reads of average length = 365 bp). Metagenomic data was assembled using Newbler 2.3 (Margulies et al., 2005), generating a 4.8 Mbp assembly in 6,113 contigs of N50 equal to 941 bp and a maximum contig size of 7,845 bp. Data are publicly available at MG-RAST (reads: mgm4454183.3; assembly: mgm4454167.3). 16S/18S rRNA gene sequence from clone libraries available in NCBI GenBank (MF360994-MF361083; MF372423-MF372506). Metagenome data is associated with NCBI BioProject number PRJNA390846 and raw sequence read data is available in the NCBI SRA (SRR5710041). For the assembled metagenome, a Whole Genome Shotgun project has been deposited at DDBJ/ENA/GenBank under the accession NJIW00000000. The version described in this paper is version NJIW01000000.

### Sequence Analysis and Phylogenetic Reconstruction

The MG-RAST 3.3 pipeline was used to classify unassembled metagenomic sequence reads as ribosomal RNA and protein coding sequences using the M5RNA and M5NR databases to provide a diversity description shown in **Figure 2** (Meyer et al., 2008). Metagenomic sequence data (both assembled sequences and unassembled reads) was mined using MG-RAST. Functions of interest were mined using the hierarchical function assignment and were assigned to GB-CYN1 when the best hits (amino acid similarity search) were to sequenced *Chroococcales* genomes. Marker genes were further investigated via profile HMM (Eddy, 2011) and BLASTX searches (Altschul et al., 1990).

PCR amplified SSU rRNA gene sequences were aligned to the SILVA 115 database (Quast et al., 2013) using SINA (Pruesse et al., 2012) and curated using ARB (Ludwig et al., 2004). 16S rRNA phylogenies were inferred using the GTRGAMMA rate approximation. For functional genes of interest that were found to have frameshift sequencing errors (*nifH, nosZ*), the sequences were corrected using Framebot (Wang et al., 2013). The metagenomic *nifH* amino acid sequence was aligned to references sequences from the *nifH* database available from the Zehr research group (Zehr and Jenkins, 2003) using the ARB software package (Ludwig et al., 2004). ProtTest3 was used to select the fixed WAG model of amino acid evolution with an inverse gamma rate approximation (Darriba et al., 2011) for the *nifH* phylogeny, though similar topologies were recovered using related models.

Phylogenetic reconstruction for both 16S rRNA and *nifH* genes were conducted as follows: maximum likelihood phylogeny was constructed with RAxML 7.2.8 (Stamatakis, 2006) with 1000 rapid bootstrap inferences and Bayesian phylogeny with MrBayes 3.3 (Ronquist et al., 2012). For Bayesian tree inferences, MCMC was run with default parameters and convergence was assessed when the reported average standard deviation of split frequencies fell below 0.01.

Hidden Markov model (HMM) profiles of 40 phylogenetic marker genes (phyeco markers) for *Bacteria* and *Archaea* (Wu et al., 2013) were used to search the green berry metagenomic peptide sequence database (from six frame translated assembled nucleotide sequence data) using the trusted cutoffs. For each of the 34 markers that have green berry hits, green berry peptide sequences were aligned with all the bacterial and archaeal references sequences by hmmalign in HMMER3 (Eddy, 2011). A maximum likelihood tree was built by Fastree2 (Price et al., 2010) for each alignments, and the alignments and trees were examined. As a result, 33 green berry peptide sequences were selected for further analysis because they branched with *Cyanobacteria* with good alignments.

These sequences were further filtered to include only those 29 phyeco gene families with a single copy, cyanobacterial green berry hit. Single-copied reference sequences from 126 *Cyanobacteria* genomes of the 29 markers and the green berry cyanobacterial metagenomic sequences were retrieved from the alignments built in the previous step and were concatenated into a large alignment. A maximum likelihood tree was built using PHYML3.0 with the LG substitution model (Guindon et al., 2010). Tree topology and branch lengths were optimized by the program and SH statistics was used for branch support estimation.

### Acetylene Reduction Assay

Whole-aggregate *in vivo* acetylene reduction assays were conducted on two 30 mL serum bottles of five washed green berries each. Acetylene, generated by the hydration of calcium carbide in an evacuated 150 mL serum-vial, was added to 10% of the headspace in a 30 mL serum-vial containing the berries in 5 mL of anoxic 0.2 μm filtered *in situ* marsh water under an 90% N_2_-CO_2_ atmosphere (90:10). Experiments were incubated on a 14 h light, 10 h dark cycle with full spectrum illumination at 30°C for 2 days. Acetylene and ethylene were quantified using a Varian 2400 series gas chromatograph (Varian Instrument Group, Walnut Creek, CA, United States) with an H_2_ flame ionization detector, as described previously (Lobo and Zinder, 1988). Rates were calculated over the 2 days incubation period with the assumption that nitrogen fixation was restricted to the dark hours of incubation, as previously described for unicellular cyanobacteria (Dron et al., 2013).

### Analysis of C:N Content

Six green berries sampled from the acetylene reduction experiment (control conditions without acetylene added) in late afternoon were dried at 55°C and crimped in aluminum foil. Samples were analyzed using a Europa 20-20 elemental analyzer interfaced with a Europa ANCA-SL mass analyzer (Sercon Limited, Cheshire, United Kingdom) under contract with the Stable Isotope Lab at the Marine Biological Laboratory’s Ecosystem Center (Woods Hole, MA, United States).

### Microelectrode Measurements

Individual aggregates were placed on an agar plate and covered with filter-sterilized seawater collected from the marsh pool. An airstream was directed at the water so that slight ripples were visible on the surface. The water had a salinity of 3.5 psu and the temperature during measurements was either 24 or 27°C, which was taken into account for calibration. We used Clark-type oxygen microsensors (tip diameter 50 μm, 90% response time < 1 s; Unisense A/S, Aarhus, Denmark), and calibrated before and after measurements in air-saturated (by bubbling) and anaerobic (0.1 M acetate, 0.1 M NaOH) 3.5% NaCl. The micro-profiling apparatus and software Sensor Trace Suite was also provided from Unisense. Starting at the water surface, microprofiles through individual aggregates were measured in the light, and light-dark shifts. Time-lapse light-dark-shift recordings included sensor tips placed directly above the aggregate (in the water phase); and inserting the tip into the central core of the aggregate. Recordings of the oxygen signal were taken every second. Light sources were 65 W halogen lamps and experiments were conducted with either 170 μmol photons m^-2^ s^-1^ (one lamp), or 320 μmol photons m^-2^ s^-1^ (two lamps). Dark conditions were realized by switching off the lamps, removing them from the table, and carefully placing a carton box over the entire profiling setup to avoid residual light from the room. Background light intensities under the box were < 1 μmol m^-2^ s^-1^.

Theoretical limits of oxygen and DIC flux and whole aggregate O_2_ flux calculations were calculated from depth concentration profiles according to Ploug et al. (1997), with a diffusion coefficient for O_2_ in 3.5% saline water of 2.175 × 10^-5^ cm^2^ s^-1^ at 24°C and 2.3535 × 10^-5^ cm^2^ s^-1^ at 27°C. Inside the aggregate, the apparent diffusivity of O_2_ was assumed to be 0.95 (Ploug and Passow, 2007). Carbon fixation was estimated based on a photosynthetic quotient of 1.2 (Masotti et al., 2007). The diffusion of oxygen in agar (0.2–2%) was not found to be different than in water over a wide range of salinities (Revsbech, 1989).

## Author Contributions

Experiments were conducted by EW, VS-C, UJ, and PH. EW analyzed data and prepared the manuscript with VS-C. JE, DB, and SZ advised and assisted with experiments and edited and revised the manuscript.

## Conflict of Interest Statement

The authors declare that the research was conducted in the absence of any commercial or financial relationships that could be construed as a potential conflict of interest.

## Supplementary Material

TABLE S1 Summary of metagenomic data for the unicellular cyanobacteria GB-CYN1. Analysis includes assignment of phylogenetic marker genes to GB-CYN1 and analysis of genes from different metabolic pathways of interest.

TABLE S2 Summary of metagenomic analysis of marker genes for nitrate and nitrite reduction.

## References

[B1] Altschul S. F. Gish W. Miller W. Myers E. W. Lipman D. J. (1990). Basic local alignment search tool. *J. Mol. Biol.* 215 403–410. 10.1016/S0022-2836(05)80360-2 2231712

[B2] Bandyopadhyay A. Elvitigala T. Welsh E. (2011). Novel metabolic attributes of the genus *Cyanothece*, comprising a group of unicellular nitrogen-fixing cyanobacteria. *mBio* 2:e00214-11 10.1128/mBio.00214-11 PMC3187577 21972240

[B3] Bench S. R. Heller P. Frank I. Arciniega M. Shilova I. N. Zehr J. P. (2013). Whole genome comparison of six *Crocosphaera watsonii* strains with differing phenotypes. *J. Phycol.* 49 786–801. 10.1111/jpy.12090 27007210 PMC5945289

[B4] Bench S. R. Ilikchyan I. N. Tripp H. J. Zehr J. P. (2011). Two strains of *Crocosphaera watsonii* with highly conserved genomes are distinguished by strain-specific features. *Front. Microbiol.* 2:261 10.3389/fmicb.2011.00261 PMC3247675 22232617

[B5] Bolhuis H. Severin I. Confurius-Guns V. Wollenzien U. I. A. Stal L. J. (2010). Horizontal transfer of the nitrogen fixation gene cluster in the cyanobacterium *Microcoleus chthonoplastes*. *ISME J.* 4 121–130. 10.1038/ismej.2009.99 19741736

[B6] Burke C. Thomas T. Lewis M. Steinberg P. Kjelleberg S. (2011). Composition, uniqueness and variability of the epiphytic bacterial community of the green alga *Ulva australis*. *ISME J.* 5 590–600. 10.1038/ismej.2010.164 21048801 PMC3105733

[B7] Cantera J. J. L. Kawasaki H. Seki T. (2004). The nitrogen-fixing gene (*nifH*) of *Rhodopseudomonas palustris*: a case of lateral gene transfer? *Microbiology* 150 2237–2246. 15256566 10.1099/mic.0.26940-0

[B8] Capone D. G. Zehr J. P. Paerl H. W. Bergman B. Carpenter E. J. (1997). *Trichodesmium*, a globally significant marine cyanobacterium. *Science* 276 1221–1229. 10.1126/science.276.5316.1221

[B9] Carpenter J. Raalte D. V. Valiela I. (1978). Nitrogen fixation by algae in a Massachusetts salt marsh. *Limnol. Oceanogr.* 23 318–327. 10.4319/lo.1978.23.2.0318

[B10] Darriba D. Taboada G. L. Doallo R. Posada D. (2011). ProtTest 3: fast selection of best-fit models of protein evolution. *Bioinformatics* 27 1164–1165. 10.1093/bioinformatics/btr088 21335321 PMC5215816

[B11] DeLong E. F. Franks D. G. Alldredge A. L. (1993). Phylogenetic diversity of aggregate-attached vs. free-living marine bacterial assemblages. *Limnol. Oceanogr.* 38 924–934. 10.4319/lo.1993.38.5.0924

[B12] Deutsch C. Sarmiento J. L. Signman D. M. Gruber N. Dunne J. P. (2007). Spatial coupling of nitrogen inputs and losses in the ocean. *Nature* 445 163–167. 10.1038/nature05392 17215838

[B13] Dron A. Rabouille S. Claquin P. Le Roy B. Talec A. Sciandra A. (2012). Light-dark (12:12) cycle of carbon and nitrogen metabolism in *Crocosphaera watsonii* WH8501: relation to the cell cycle. *Environ. Microbiol.* 14 967–981. 10.1111/j.1462-2920.2011.02675.x 22188053

[B14] Dron A. Rabouille S. Claquin P. Talec A. Raimbault V. Sciandra A. (2013). Photoperiod length paces the temporal orchestration of cell cycle and carbon-nitrogen metabolism in *Crocosphaera watsonii*. *Environ. Microbiol.* 15 3292–3304. 10.1111/1462-2920.12163 23841885

[B15] Eddy S. R. (2011). Accelerated profile HMM searches. *PLoS Comput. Biol.* 7:e1002195 10.1371/journal.pcbi.1002195 PMC3197634 22039361

[B16] Eloe E. A. Shulse C. N. Fadrosh D. W. Williamson S. J. Allen E. E. Bartlett D. H. (2011). Compositional differences in particle-associated and free-living microbial assemblages from an extreme deep-ocean environment. *Environ. Microbiol. Rep.* 3 449–458. 10.1111/j.1758-2229.2010.00223.x 23761307

[B17] Fernandes N. Steinberg P. Rusch D. Kjelleberg S. Thomas T. (2012). Community structure and functional gene profile of bacteria on healthy and diseased thalli of the red seaweed *Delisea pulchra*. *PLoS ONE* 7:e50854 10.1371/journal.pone.0050854 PMC3513314 23226544

[B18] Foster R. A. Subramaniam A. Mahaffey C. Carpenter E. J. Capone D. G. Zehr J. P. (2007). Influence of the Amazon River plume on distributions of free-living and symbiotic cyanobacteria in the western tropical north Atlantic Ocean. *Limnol. Oceanogr.* 52 517–532. 10.4319/lo.2007.52.2.0517

[B19] Foster R. A. Zehr J. P. (2006). Characterization of diatom-cyanobacteria symbioses on the basis of *nifH, hetR* and 16S rRNA sequences. *Environ. Microbiol.* 8 1913–1925. 10.1111/j.1462-2920.2006.01068.x 17014491

[B20] Fulweiler R. W. Brown S. M. Nixon S. W. Jenkins B. D. (2013). Evidence and a conceptual model for the co-occurrence of nitrogen fixation and denitrification in heterotrophic marine sediments. *Mar. Ecol. Prog. Ser.* 482 57–68. 10.3354/meps10240

[B21] Gaby J. C. Buckley D. H. (2014). A comprehensive aligned nifH gene database: a multipurpose tool for studies of nitrogen-fixing bacteria. *Database* 2014:bau001 10.1093/database/bau001 PMC3915025 24501396

[B22] Galloway J. N. Dentener F. J. Capone D. G. Boyer E. W. Howarth R. W. Seitzinger S. P. (2004). Nitrogen cycles: past, present, and future. *Biogeochemistry* 70 153–226. 10.1007/s10533-004-0370-0

[B23] Guindon S. Dufayard J. F. Lefort V. Anisimova M. Hordijk W. Gascuel O. (2010). New algorithms and methods to estimate maximum-likelihood phylogenies: assessing the performance of PhyML 3.0. *Syst. Biol.* 59 307–321. 10.1093/sysbio/syq010 20525638

[B24] Hagino K. Onuma R. Kawachi M. Horiguchi T. (2013). Discovery of an endosymbiotic nitrogen-fixing cyanobacterium UCYN-A in *Braarudosphaera bigelowii* (*Prymnesiophyceae*). *PLoS ONE* 8:e81749 10.1371/journal.pone.0081749 PMC3852252 24324722

[B25] Hewson I. Poretsky R. S. Dyhrman S. T. Zielinski B. White A. E. Tripp H. J. (2009). Microbial community gene expression within colonies of the diazotroph, *Trichodesmium*, from the Southwest Pacific Ocean. *ISME J.* 3 1286–1300. 10.1038/ismej.2009.75 19571897

[B26] Hietanen S. Moisander P. H. Kuparinen J. Tuominen L. (2002). No sign of denitrification in a Baltic Sea cyanobacterial bloom. *Mar. Ecol. Prog. Ser.* 242 73–82. 10.3354/meps242073

[B27] Howarth R. W. Marina R. Lane J. Cole J. J. (1988). Nitrogen fixation in freshwater, estuarine, and marine ecosystems. 1. Rates and importance. *Limnol. Oceanogr.* 33 669–687. 10.4319/lo.1988.33.4_part_2.0669

[B28] Karl D. M. Michaels A. Bergman B. Capone D. G. Carpenter E. J. Letelier R. (2002). Dinitrogen fixation in the world’s oceans. *Biogeochemistry* 5 47–98. 10.1023/A:1015798105851

[B29] Klawonn I. Bonaglia S. Brüchert V. Ploug H. (2015). Aerobic and anaerobic nitrogen transformation processes in N_2_-fixing cyanobacterial aggregates. *ISME J.* 9 1456–1466. 10.1038/ismej.2014.232 25575306 PMC4438332

[B30] Langlois R. J. Laroche J. Raab P. A. (2005). Diazotrophic diversity and distribution in the tropical and subtropical Atlantic Ocean. *Appl. Environ. Microbiol.* 71 7910–7919. 10.1128/AEM.71.12.7910-7919.2005 16332767 PMC1317331

[B31] Lobo A. L. Zinder S. H. (1988). Diazotrophy and nitrogenase activity in the archaebacterium *Methanosarcina barkeri* 227. *Appl. Environ. Microbiol.* 54 1656–1661. 16347675 10.1128/aem.54.7.1656-1661.1988 PMC202723

[B32] Ludwig W. Strunk O. Westram R. Richter L. Meier H. Yadhukumar (2004). ARB: a software environment for sequence data. *Nucleic Acids Res.* 32 1363–1371. 10.1093/nar/gkh293 14985472 PMC390282

[B33] Mann E. L. Barbeau K. A. (2014). *Data from: Roseibium sp. TrichSKD4: GOLD Card Gi03577. Genomes Online Database.* (2014). Available at: http://genomesonline.org/cgi-bin/GOLD/bin/GOLDCards.cgi?goldstamp=Gi03577

[B34] Margulies M. Egholm M. Altman W. E. Attiya S. Bader J. S. Bemben L. A. (2005). Genome sequencing in microfabricated high-density picolitre reactors. *Nature* 437 376–380. 10.1038/nature03959 16056220 PMC1464427

[B35] Masotti I. Ruiz Pino D. Le Bouteiller A. (2007). Photosynthetic characteristics of *Trichodesmium* in the southwest Pacific Ocean: importance and significance. *Mar. Ecol. Prog. Ser.* 338 47–59. 10.3354/meps338047

[B36] Meyer F. Paarmann D. D’souza M. Olson R. Glass E. M. Kubal M. (2008). The metagenomics RAST server – a public resource for the automatic phylogenetic and functional analysis of metagenomes. *BMC Bioinformatics* 9:386 10.1186/1471-2105-9-386 PMC2563014 18803844

[B37] Meyer J. L. Huber J. A. (2014). Strain-level genomic variation in natural populations of *Lebetimonas* from an erupting deep-sea volcano. *ISME J.* 8 867–880. 10.1038/ismej.2013.206 24257443 PMC3960544

[B38] Montoya J. P. Holl C. M. Zehr J. P. Hansen A. Villareal T. A. Capone D. G. (2004). High rates of N_2_ fixation by unicellular diazotrophs in the oligotrophic Pacific Ocean. *Nature* 430 1027–1031. 10.1038/nature02824 15329721

[B39] Mota R. Guimarães R. Büttel Z. Rossi F. Colica G. Silva C. J. (2013). Production and characterization of extracellular carbohydrate polymer from *Cyanothece* sp. CCY 0110. *Carbohydr. Polym.* 92 1408–1415. 10.1016/j.carbpol.2012.10.070 23399171

[B40] Paerl H. W. Bebout B. M. (1988). Direct measurement of O_2_-depleted microzones in marine *Oscillatoria*: relation to N_2_ fixation. *Science* 241 442–445. 10.1126/science.241.4864.442 17792609

[B41] Paerl H. W. Bebout B. M. Prufert L. E. (1989). Bacterial associations with marine *Oscillatoria* sp. (*Trichodesmium* sp.) populations: ecophysiological implications. *J. Phycol.* 25 773–784. 10.1111/j.0022-3646.1989.00773.x

[B42] Paerl H. W. Prufert L. E. (1987). Oxygen-poor microzones as potential sites of microbial N_2_ fixation in nitrogen-depleted aerobic marine waters. *Appl. Environ. Microbiol.* 53 1078–1087. 16347337 10.1128/aem.53.5.1078-1087.1987 PMC203813

[B43] Parker M. A. (2012). Legumes select symbiosis island sequence variants in *Bradyrhizobium*. *Mol. Ecol.* 21 1769–1778. 10.1111/j.1365-294X.2012.05497.x 22369247

[B44] Passow U. (2002). Transparent exopolymer particles (TEP) in aquatic environments. *Prog. Oceanogr.* 55 287–333. 10.1016/S0079-6611(02)00138-6

[B45] Pereira S. Zille A. Micheletti E. Moradas-Ferreira P. De Philippis R. Tamagnini P. (2009). Complexity of cyanobacterial exopolysaccharides: composition, structures, inducing factors and putative genes involved in their biosynthesis and assembly. *FEMS Microbiol. Rev.* 33 917–941. 10.1111/j.1574-6976.2009.00183.x 19453747

[B46] Ploug H. (2008). Cyanobacterial surface blooms formed by *Aphanizomenon* sp. and *Nodularia spumigena* in the Baltic Sea: small-scale fluxes, pH, and oxygen microenvironments. *Limnol. Oceanogr.* 53 914–921. 10.4319/lo.2008.53.3.0914

[B47] Ploug H. Adam B. Musat N. Kalvelage T. Lavik G. Wolf-Gladrow D. (2011). Carbon, nitrogen and O_2_ fluxes associated with the cyanobacterium *Nodularia spumigena* in the Baltic Sea. *ISME J.* 5 1549–1558. 10.1038/ismej.2011.20 21390075 PMC3160678

[B48] Ploug H. Kuhl M. Buchholz-Cleven B. Jørgensen B. B. (1997). Anoxic aggregates - an ephemeral phenomenon in the pelagic environment? *Aquat. Microb. Ecol.* 13 285–294. 10.3354/ame013285

[B49] Ploug H. Musat N. Adam B. Moraru C. L. Lavik G. Vagner T. (2010). Carbon and nitrogen fluxes associated with the cyanobacterium *Aphanizomenon* sp. in the Baltic Sea. *ISME J.* 4 1215–1223. 10.1038/ismej.2010.53 20428225

[B50] Ploug H. Passow U. (2007). Direct measurement of diffusivity within diatom aggregates containing transparent exopolymer particles. *Limnol. Oceanogr.* 52 1–6. 10.4319/lo.2007.52.1.0001

[B51] Price M. N. Dehal P. S. Arkin A. P. (2010). FastTree 2 - approximately maximum-likelihood trees for large alignments. *PLoS ONE* 5:e9490 10.1371/journal.pone.0009490 PMC2835736 20224823

[B52] Pruesse E. Peplies J. Glockner F. O. (2012). SINA: accurate high-throughput multiple sequence alignment of ribosomal RNA genes. *Bioinformatics* 28 1823–1829. 10.1093/bioinformatics/bts252 22556368 PMC3389763

[B53] Quast C. Pruesse E. Yilmaz P. Gerken J. Schweer T. Yarza P. (2013). The SILVA ribosomal RNA gene database project: improved data processing and web-based tools. *Nucleic Acids Res.* 41 D590–D596. 10.1093/nar/gks1219 23193283 PMC3531112

[B54] Reddy K. J. Haskell J. B. Sherman D. M. Sherman L. A. (1993). Unicellular, aerobic nitrogen-fixing cyanobacteria of the genus *Cyanothece*. *J. Bacteriol.* 175 1284–1292. 10.1128/jb.175.5.1284-1292.1993 8444791 PMC193213

[B55] Redfield A. C. Ketchum B. H. Richards F. A. (1963). “The influence of organisms on the composition of sea-water,” in *The Sea* ed. Hill M. N. (New York, NY: Wiley-Interscience) 26–77.

[B56] Revsbech N. P. (1989). Diffusion characteristics of microbial communities determined by use of oxygen microsensors. *J. Microbiol. Methods* 9 111–122. 10.1016/0167-7012(89)90061-4

[B57] Revsbech N. P. Jørgensen B. B. Brix O. (1981). Primary production of microalgae in sediments measured by oxygen microprofile, H_14_CO_3_- fixation, and oxygen exchange methods. *Limnol. Oceanogr.* 26 717–730. 10.4319/lo.1981.26.4.0717

[B58] Ronquist F. Teslenko M. Van Der Mark P. Ayres D. L. Darling A. Hohna S. (2012). MrBayes 3.2: efficient Bayesian phylogenetic inference and model choice across a large model space. *Syst. Biol.* 61 539–542. 10.1093/sysbio/sys029 22357727 PMC3329765

[B59] Seitz A. P. Nielsen T. H. Overmann J. (1993). Physiology of purple sulfur bacteria forming macroscopic aggregates in Great Sippewissett Salt Marsh, Massachusetts. *FEMS Microbiol. Ecol.* 12 225–235. 10.1111/j.1574-6941.1993.tb00035.x

[B60] Shapiro O. H. Hatzenpichler R. Buckley D. H. Zinder S. H. Orphan V. J. (2011). Multicellular photo-magnetotactic bacteria. *Environ. Microbiol. Rep.* 3 233–238. 10.1111/j.1758-2229.2010.00215.x 23761255

[B61] Sohm J. A. Edwards B. R. Wilson B. G. Webb E. A. (2011). Constitutive extracellular polysaccharide (EPS) production by specific isolates of *Crocosphaera watsonii*. *Front. Microbiol.* 2:229–229. 10.3389/fmicb.2011.00229 22110469 PMC3215947

[B62] Stamatakis A. (2006). RAxML-VI-HPC: maximum likelihood-based phylogenetic analyses with thousands of taxa and mixed models. *Bioinformatics* 22 2688–2690. 10.1093/bioinformatics/btl446 16928733

[B63] Thompson A. W. Foster R. A. Krupke A. Carter B. J. Musat N. Vaulot D. (2012). Unicellular cyanobacterium symbiotic with a single-celled eukaryotic alga. *Science* 337 1546–1550. 10.1126/science.1222700 22997339

[B64] Tripp H. J. Bench S. R. Turk K. A. Foster R. A. Desany B. A. Niazi F. (2010). Metabolic streamlining in an open-ocean nitrogen-fixing cyanobacterium. *Nature* 464 90–94. 10.1038/nature08786 20173737

[B65] Tuomainen J. Hietanen S. Kuparinen J. Martikainen P. J. Servomaa K. (2006). Community structure of the bacteria associated with *Nodularia* sp. (*Cyanobacteria*) aggregates in the Baltic Sea. *Microb. Ecol.* 52 513–522. 10.1007/s00248-006-9130-0 16944338

[B66] Tuomainen J. M. Hietanen S. Kuparinen J. Martikainen P. J. Servomaa K. (2003). Baltic Sea cyanobacterial bloom contains denitrification and nitrification genes, but has negligible denitrification activity. *FEMS Microbiol. Ecol.* 45 83–96. 10.1016/S0168-6496(03)00131-4 19719619

[B67] Wang Q. Quensen Iii J. F. Fish J. A. Lee T. K. Sun Y. Tiedje J. M. (2013). Ecological patterns of *nifH* genes in four terrestrial climatic zones explored with targeted metagenomics using FrameBot, a new informatics tool. *mBio* 4:e00592-13 10.1128/mBio.00592-13 PMC3781835 24045641

[B68] Webb E. A. Ehrenreich I. M. Brown S. L. Valois F. W. Waterbury J. B. (2009). Phenotypic and genotypic characterization of multiple strains of the diazotrophic cyanobacterium, *Crocosphaera watsonii*, isolated from the open ocean. *Environ. Microbiol.* 11 338–348. 10.1111/j.1462-2920.2008.01771.x 19196268

[B69] Welsh E. A. Liberton M. Stöckel J. Loh T. Elvitigala T. Wang C. (2008). The genome of *Cyanothece* 51142, a unicellular diazotrophic cyanobacterium important in the marine nitrogen cycle. *Proc. Natl. Acad. Sci. U.S.A.* 105 15094–15099. 10.1073/pnas.0805418105 18812508 PMC2567498

[B70] Wilbanks E. G. Jaekel U. Salman V. Humphrey P. T. Eisen J. A. Faccioti M. T. (2014). Microscale sulfur cycling in the phototrophic pink berry consortia of the Sippewissett Salt Marsh. *Environ. Microbiol.* 16 3398–3415. 10.1111/1462-2920.12388 24428801 PMC4262008

[B71] Wu D. Jospin G. Eisen J. A. (2013). Systematic identification of gene families for use as “markers” for phylogenetic and phylogeny-driven ecological studies of bacteria and archaea and their major subgroups. *PLoS ONE* 8:e77033 10.1371/journal.pone.0077033 PMC3798382 24146954

[B72] Wyman M. Hodgson S. Bird C. (2013). Denitrifying alphaproteobacteria from the Arabian Sea that express *nosZ*, the gene encoding nitrous oxide reductase, in oxic and suboxic waters. *Appl. Environ. Microbiol.* 79 2670–2681. 10.1128/AEM.03705-12 23396348 PMC3623181

[B73] Zehr J. P. Bench S. R. Carter B. J. Hewson I. Niazi F. Shi T. (2008). Globally distributed uncultivated oceanic N_2_-fixing cyanobacteria lack oxygenic photosystem II. *Science* 322 1110–1112. 10.1126/science.1165340 19008448

[B74] Zehr J. P. Jenkins B. D. (2003). Nitrogenase gene diversity and microbial community structure: a cross-system comparison. *Environ. Microbiol.* 5 539–554. 10.1046/j.1462-2920.2003.00451.x 12823187

[B75] Zehr J. P. Montoya J. P. Jenkins B. D. Hewson I. Mondragon E. Short C. M. (2007). Experiments linking nitrogenase gene expression to nitrogen fixation in the North Pacific subtropical gyre. *Limnol. Oceanogr.* 52 169–183. 10.4319/lo.2007.52.1.0169

[B76] Zehr J. P. Waterbury J. B. Turner P. J. Montoya J. P. Omoregie E. Steward G. F. (2001). Unicellular cyanobacteria fix N_2_ in the subtropical North Pacific Ocean. *Nature* 715 25–28. 10.1038/35088063 11493920

