# Supplementary material for: The Green Berry Consortia of the Sippewissett Salt Marsh: Millimeter-Sized Aggregates of Diazotrophic Unicellular Cyanobacteria
Source: Front Microbiol. 2017 Sep 4;8:1623. doi: 10.3389/fmicb.2017.01623 (PMC5591377; doi:10.3389/fmicb.2017.01623)
Supplement: Supplementary file 3 [file Image_1.PDF]

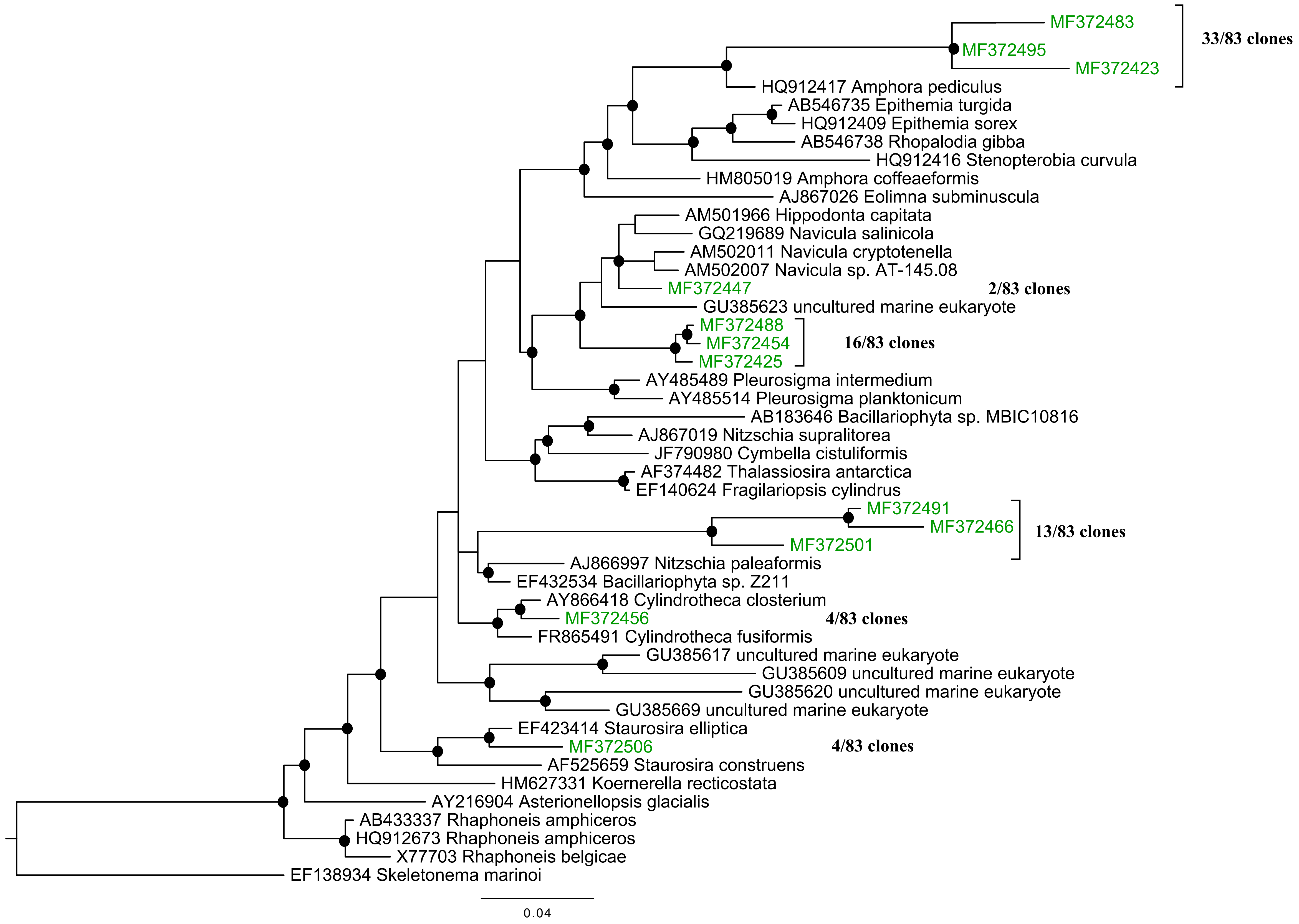

Supplemental Figure 1. 18S rRNA gene phylogeny of 97% operational taxonomic units (OTUs) from the green berry consortia (shown in green, ~700 bp PCR amplified sequences) and reference sequences from within the *Bacillariophyta* (diatoms). Nodes strongly supported by both maximum likelihood (1000 bootstrap replicates, support >50%) and Bayesian tree inferences (posterior probability >0.75) are marked by a dot. The number of clone sequences from the 18S rRNA clone library that belong to different OTU clades is shown in bold to the right of the sequences. Scale bar represents the mean number of nucleotide substitutions per site.
