# Supplementary material for: The Green Berry Consortia of the Sippewissett Salt Marsh: Millimeter-Sized Aggregates of Diazotrophic Unicellular Cyanobacteria
Source: Front Microbiol. 2017 Sep 4;8:1623. doi: 10.3389/fmicb.2017.01623 (PMC5591377; doi:10.3389/fmicb.2017.01623)

Supplemental Figure 2. Maximum likelihood phylogenic tree from the concatenated alignment of 29 phyeco marker genes from 126 single-copy cyanobacterial reference genomes and single-copy green berry cyanobacterial metagenomic sequences. The tree was built using PHYML3.0 with the LG substitution model with topology and branch lengths optimized by the program and SH statistics (shown at the nodes) was used for branch support estimation. Numbers at the beginning of taxon names are NCBI UID for the genome preceded by the letter B (Bacteria). Scale bar represents mean amino acid substitutions per site.

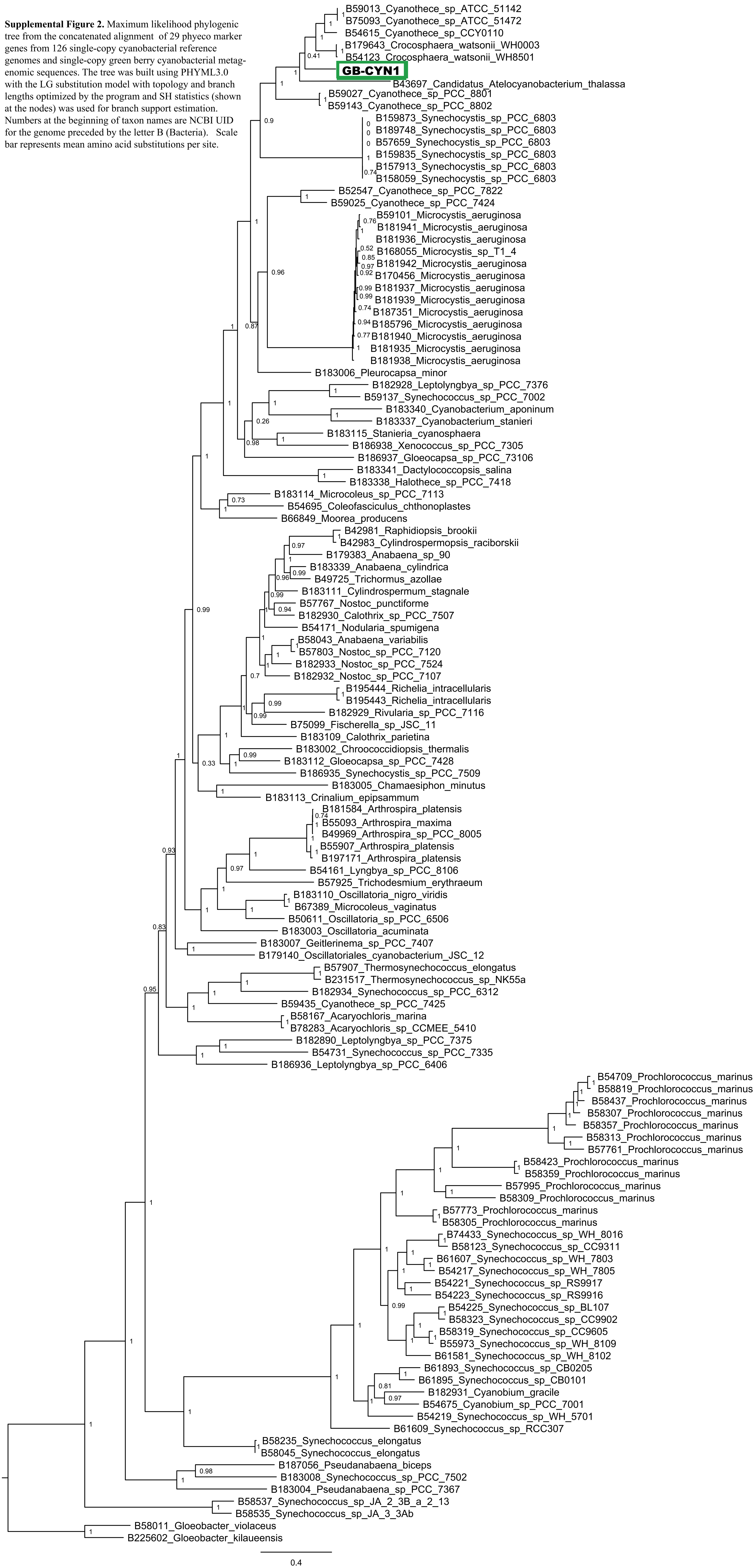

Supplement: Supplementary file 4 [file Image_2.PDF]
